# Internet‐delivered therapist‐assisted cognitive therapy for adolescent social anxiety disorder (OSCA): a randomised controlled trial addressing preliminary efficacy and mechanisms of action

**DOI:** 10.1111/jcpp.13680

**Published:** 2022-08-09

**Authors:** Eleanor Leigh, David M. Clark

**Affiliations:** ^1^ Department of Experimental Psychology University of Oxford Oxford UK; ^2^ Oxford Health NHS Foundation Trust Oxford UK

**Keywords:** Social anxiety disorder, adolescent, CBT, cognitive therapy, internet, digital

## Abstract

**Background:**

Cognitive therapy for SAD (CT‐SAD) is a first‐line recommended treatment for adult social anxiety disorder (SAD) and shows considerable promise for youth. However, the high prevalence of adolescent SAD and limited number of therapists presents an implementation challenge. Delivery of CT‐SAD via the Internet may offer part of the solution.

**Method:**

Forty‐three youth (14–18 years) with SAD recruited through schools were randomly allocated to therapist‐assisted Internet‐delivered CT‐SAD (called OSCA) or waitlist for 14 weeks (ISRCTN15079139).

**Results:**

OSCA outperformed waitlist on all measures and was associated with large effects that were maintained at 6‐month follow‐up. In the OSCA arm, 77% of adolescents lost their SAD diagnosis at post (vs. 14% in the waitlist arm), increasing to 91% at 6‐months. Beneficial effects of OSCA were mediated through changes in cognitions and safety behaviours as predicted by cognitive models of SAD. OSCA was associated with high credibility and therapeutic alliance.

**Conclusions:**

This preliminary trial suggests OSCA holds promise as an effective, accessible treatment for adolescent SAD. Future definitive trials could compare OSCA to active comparators to examine specificity of effects.

## Introduction

One of the most common mental health problems in adolescence is social anxiety disorder (SAD), affecting 2 to 3 pupils in every classroom (Fehm, Pelissolo, Furmark, & Wittchen, 2005; Wittchen, Stein, & Kessler, 1999). It rarely remits spontaneously (Bruce et al., 2005) and carries with it a risk of further anxiety and depressive disorders (Stein et al., 2001), suicidality and functional impairment (Chiu, Clark, & Leigh, 2021b; Vilaplana‐Pérez et al., 2020).

Despite the need for evidence‐based and accessible treatment for adolescent SAD, it has proven difficult to treat effectively with available interventions. For example, the most widely used psychological therapy for youth anxiety disorders is a generic form of CBT designed to treat all common anxiety presentations. However, a meta‐analysis demonstrated that youth with SAD were almost 50% less likely to remit from their primary diagnosis after this type of treatment compared to those with other anxiety disorders (Evans, Clark, & Leigh, 2021). These findings have led to renewed efforts to develop SAD‐specific interventions (Lassen, Hougaard, Arendt, & Thastum, 2019). In this context, the notably large effect sizes associated with certain treatments for adult SAD are of interest. For example, cognitive therapy for SAD (CT‐SAD), based on the Clark and Wells (1995) model, stands out by its superiority to other active treatments (Mayo‐Wilson et al., 2014) and it is a first‐line NICE recommended treatment (National Institute for Health and Care Excellence, 2013). CT‐SAD has been adapted for youth and it is associated with large effects compared to standard generic CBT (Ingul, Aune, & Nordahl, 2014). CT‐SAD was designed to reverse the cognitive and behavioural processes specified in the Clark and Wells (1995) model of social anxiety. According to this model, social anxiety is driven by excessively negative beliefs about social interactions, which are maintained by self‐focused attention, dysfunctional safety behaviours and negative self‐imagery. The treatment involves procedures that specifically target each of these processes, including: attention training to promote an external focus of attention, video feedback to update negative images and behavioural experiments involving dropping safety behaviours to help individuals discover that they are more acceptable than they think.

However, substantial challenges exist to implementing time‐intensive interventions, such as CT‐SAD, in routine services due to limited availability of therapists and high prevalence of SAD. Internet delivery may offer a part of the solution. Three RCTs have evaluated Internet‐delivered SAD‐specific CBT for adolescents (Nordh et al., 2021; Spence, Donovan, March, Kenardy, & Hearn, 2017; Tillfors et al., 2011). A small waitlist‐controlled trial (*n* = 18) of Internet self‐help CBT for public‐speaking anxiety found a large effect on social anxiety symptoms (*d* = 1.28), maintained at 12‐month follow‐up (Tillfors et al., 2011). However, the low symptom levels at baseline suggest we should be cautious in generalising findings to a routinely referred clinical population. More recently, a RCT with children and adolescents (*n* = 125) compared Internet‐delivered generic CBT and SAD‐specific CBT to waitlist (Spence et al., 2017). Whilst both treatment conditions outperformed waitlist, the majority of participants in both treatment arms still met diagnostic criteria for SAD post‐treatment [remission: 13% (SAD‐focused CBT); 15% (generic CBT)]. Finally, Internet‐delivered CBT for SAD was compared with Internet‐delivered supportive therapy in 103 Swedish adolescents (Nordh et al., 2021). There was a significant between‐group effect size of *d* = 0.67 at follow‐up on social anxiety symptoms, but the proportion of youth in remission was low and did not differ between arms [31% (CBT) vs. 18% (supportive therapy)]. The findings from these three trials indicate promise for Internet‐delivered SAD‐specific CBT, but also scope for enhancing treatment outcomes further.

An Internet‐delivered therapist‐assisted version of CT‐SAD has been developed for adults and in a recent trial it has been shown to have similar effects to face‐to‐face treatment, but requiring 70% less therapist time (Clark et al., in press). The question arises as to whether Internet CT‐SAD could be beneficial for adolescents and potentially reduce demand on services, but no studies have examined this yet.

### Aims

This trial aimed to examine preliminary effectiveness of a minimally adapted version of Internet‐delivered therapist‐assisted CT‐SAD for adolescents [which we called ‘Online Social anxiety Cognitive therapy for Adolescents’ (OSCA)]. We chose a waitlist control because this is the first trial of the intervention in adolescents. We were interested in effects on social anxiety symptoms and diagnosis, as well as comorbid symptoms and functioning. An exploratory aim was to establish whether OSCA exerts its effects via the psychological mechanisms predicted by Clark & Wells' model (Clark & Wells, 1995). Finally, we were interested to learn whether OSCA is a credible treatment, whether a good therapeutic alliance can be established in the treatment, and whether either of these aspects of therapy are associated with outcome.

We hypothesised: (a) OSCA will be superior to waitlist in terms of social anxiety symptoms and diagnosis (primary outcomes); (b) gains will be maintained at follow‐up; (c) OSCA will be superior to waitlist in reducing general anxiety, depression and functioning, and parent‐reported internalising symptoms and impairment (secondary outcomes); (d) social anxiety‐related cognitions, attitudes and safety behaviours depression will mediate therapeutic improvement.

## Methods

### Design

Prospectively registered (http://www.isrctn.com/ISRCTN15079139) and ethically approved (R60464/RE001) parallel RCT with published protocol (Leigh & Clark, 2019).

### Participants

Participants were selected via screening in four secondary schools in the Southeast of England (covering a broad socioeconomic range), March 2019–December 2020 (details of COVID‐19 disruption in Appendix S1). Inclusion criteria were being aged 14–18 years with a primary diagnosis of DSM‐5 SAD. Exclusion criteria were a diagnosis of autism, learning disability, psychosis, current alcohol or substance dependence, previous receipt of CT/CBT for SAD, suicidal intent or recurrent self‐harm, or active safeguarding concerns.

### Procedure

Participants were randomised to OSCA or waitlist with minimisation to ensure balance between arms for gender and school with stratification by SAD severity [ADIS‐C/P clinical severity rating (Masia‐Warner, Fisher, Shrout, Rathor, & Klein, 2007)].

Measures were collected pre‐treatment/wait, mid‐treatment/wait (week 8) and post‐treatment/wait (week 15). Participants in the OSCA arm were also assessed weekly during treatment and at 1‐, 2‐ and 3‐ and 6‐month follow‐up.

The OSCA programme uses the same Internet platform as adult Internet CT‐SAD with minor adaptations for adolescents (see the Supporting Information). OSCA takes 14 weeks. All users receive a set of eight core modules (Table S1) to work through in the first two weeks. Then, up to 16 additional modules focusing on particular fears or problems can be released to individualise the programme for each user. The programme includes a secure video conferencing facility with recording functionality to support online delivery of two core CT‐SAD procedures: the self‐focused attention and safety behaviour experiment and video feedback. Adolescents can logon as often as they like. During the 14 weeks, young people allocated to OSCA have weekly 20‐min phone calls with their therapist and they also receive regular encouragement and support via secure messaging within the programme and SMS texts. Messages are individualised to the user, and in addition to encouragement and support, they provide reminders to engage in therapy tasks, and summaries of telephone calls. Treatment was delivered by a clinical psychologist (EL) with fortnightly supervision provided by DMC.

For waitlist participants, the only contact during the wait period was mid‐wait, when they were asked to complete outcome measures. They were offered OSCA post‐wait.

### Measures

See Table S2 for schedule and reliability of measures.

#### Primary outcome measures

Liebowitz Social Anxiety Scale for Children and Adolescents‐ Self‐report Version (LSAS‐CA‐SR) (Leigh & Clark, 2021b; Masia‐Warner, Klein, & Liebowitz, 1999) yields a continuous measure of social anxiety. Anxiety Disorders Interview Schedule IV for Children and Parents (ADIS‐C/P) (Silverman & Albano, 1996) was completed by an assessor blind to allocation to assess SAD diagnosis at pre and post (and 6‐month follow‐up for those in OSCA arm).

#### Secondary outcome measures

Child & Adolescent Social Phobia Weekly Summary Scale (Clark, 2003) measures aspects of social anxiety including avoidance and rumination (range: 0–8). Internalising symptoms assessed by self‐ and parent‐reported RCADS (Chorpita, Yim, Moffitt, Umemoto, & Francis, 2000) and Short Mood & Feelings Questionnaire (SMFQ) (Angold et al., 1995). Peer functioning assessed with Social Satisfaction Scale (total: 4–28) and Social Participation Scale (range 13–91). Self‐reported ability to concentrate in class was assessed on a single‐item scale (0–8) (Leigh & Clark, 2016). Self‐ and parent‐reported CALIS (Lyneham et al., 2013) assesses anxiety‐related impairment [range: 0–36 (self); 0–72 (parent)].

#### Measures of CT‐SAD processes

Child and Adolescent Social Cognitions Questionnaire (CASCQ) measures social anxiety‐related cognitions (Leigh & Clark, 2021a) (Frequency, range: 1–5; Belief, range: 0–100). Child and Adolescent Social Behaviour Questionnaire (CASBQ) measures safety behaviours (Chiu, Clark, & Leigh, 2021a; Evans, Chiu, Clark, Waite, & Leigh, 2021) (range: 0–3). Child and Adolescent Social Attitudes Questionnaire (CASAQ) (Leigh & Clark, 2016) is a measure of beliefs (range: 1–7).

#### Measure of therapeutic alliance and treatment credibility

Alliance and credibility were measured after two weeks of OSCA to ensure participants had received enough treatment to provide meaningful ratings but not so much as for these to be affected by treatment outcome. OSCA participants and therapists completed the Shortened Working Alliance Inventory (WAI) (Hatcher & Gillaspy, 2006) (range: 12–84). Participants completed the Credibility of Therapy Scale (Borkovec & Nau, 1972) (range: 1–10).

#### Adverse events

Adverse events were monitored and recorded from randomisation to final follow‐up.

### Analysis

Sample size estimation was based on a conservative effect size of 1, derived from Ingul et al. (2014). Seventeen per group would provide 80% power to detect a group difference with α = .05. This was inflated to 20 per group to allow for drop‐out (Smith et al., 2015).

Nuffield Primary Care Clinical Trials Unit provided statistical advice. Analyses performed in R (R Core Team, 2020) on the intention‐to‐treat sample unless specified. Linear mixed effect models were used to analyse continuous variables over time with restricted maximum likelihood estimation. Time and condition plus their interaction were specified as fixed effects, with baseline LSAS‐CA‐SR score and gender as fixed covariates and participant as a random effect. As there were two primary outcome measures, a *p* value of .025 was used for significance testing. For secondary outcome measures, the baseline score of the measure being analysed was additionally included as a fixed covariate. Normality of residuals assumption was checked using *Q*–*Q* plots and met for all models. Diagnostic status was analysed using mixed effects logistic regression, with condition and gender.

Linear mixed models with participants in the OSCA arm were used to assess maintenance of gains at 3‐ and 6‐months. Time (baseline/post/3‐month/6‐month) was included as a fixed effect, participant as a random effect and gender as a fixed covariate. Where the effect of time was significant, pairwise comparisons with a Bonferroni adjustment were used.

To assess whether treatment credibility or therapeutic alliance (participant‐ and therapist‐rated) predicted outcome, these variables were entered into a linear regression predicting post‐treatment LSAS‐CA‐SR scores. Baseline LSAS‐CA‐SR and gender were entered in the first step, followed by the alliance and credibility variables.

A model‐based inference approach to causal mediation analysis was used (Imai, Keele, & Tingley, 2010) with complete cases. Candidate mediators were social anxiety‐related cognitions (CASCQ, frequency and belief ratings), attitudes (CASAQ) and safety behaviours (CASBQ). Mid scores were used as mediator, and post LSAS‐CA‐SR scores as outcome. Baseline LSAS‐CA‐SR, gender and baseline mediator scores were included as covariates. The average causal mediation effect (ACME), average direct effect, the average total effect and percentage mediated were calculated.

## Results

### Participant flow and demographics

**Figure 1:**
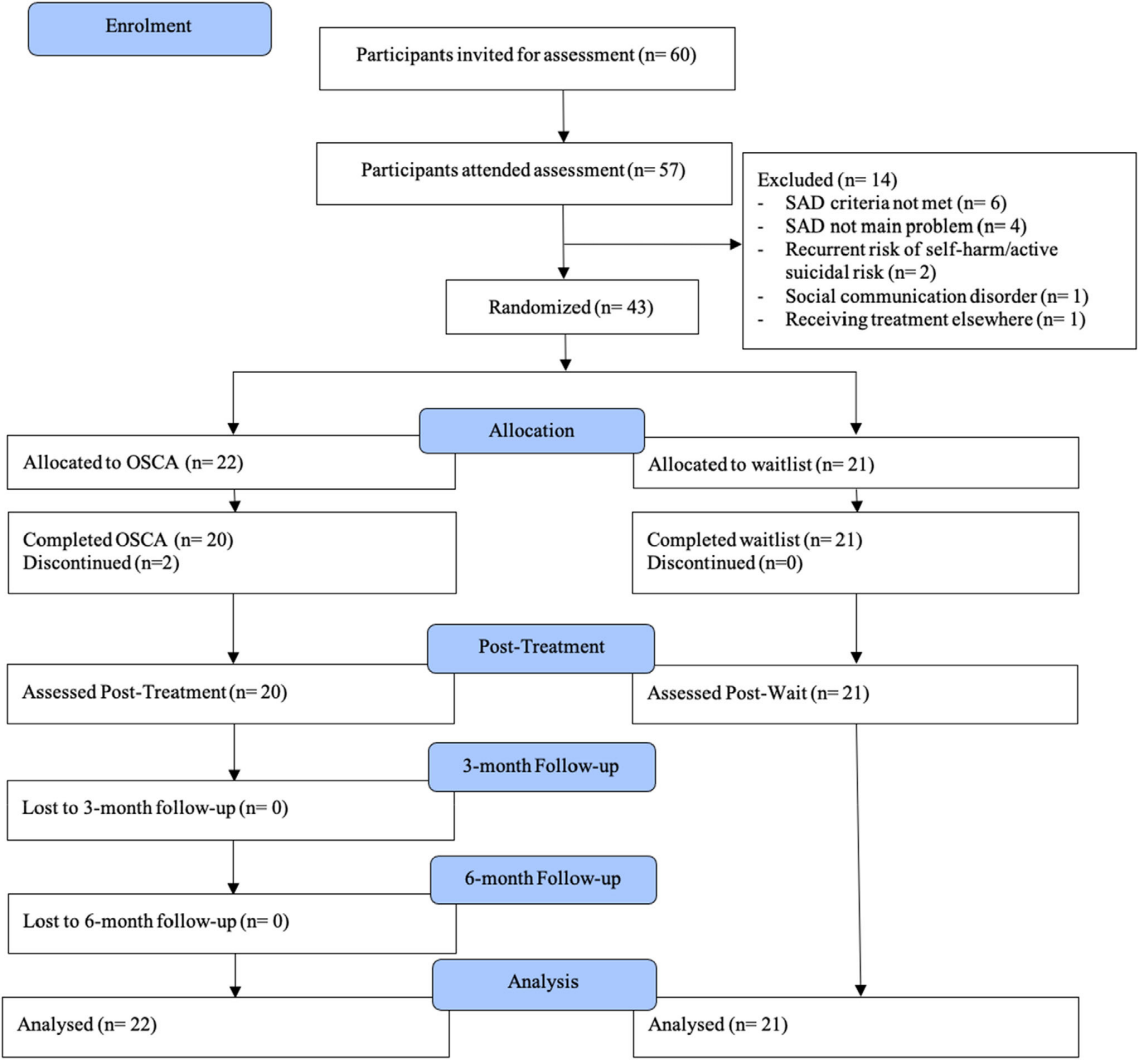
Participant flow through the trial [Color figure can be viewed at wileyonlinelibrary.com]

Figure 1 shows the CONSORT flow diagram. Demographic and clinical characteristics are shown in Table 1. The total proportion of participants from minority ethnic backgrounds (48.8%) in the sample was greater than the average in England (33.2% in 2019/2020).

**Table 1:** Baseline demographic and clinical characteristics

	Waitlist (*N* = 21)	OSCA (*N* = 22)	Total (*N* = 43)
Age [years; *M* (*SD*)]	16.42 (1.11)	16.04 (1.03)	16.22 (1.08)
Female [*N* (%)]	19 (90%)	20 (91%)	39 (91%)
Historical peer victimisation [*N* (%)]	6 (29%)	5 (23%)	11 (26%)
On‐going peer victimisation [*N* (%)]	6 (29%)	4 (18%)	10 (23%)
Previous psychological therapy [*N* (%)]	9 (43%)	5 (23%)	14 (33%)
ADIS CSR for SAD diagnosis [0–8; *M* (*SD*)]	5.62 (1.28)	5.23 (0.97)	5.42 (1.14)
Comorbidity [*N* (%)]	13 (62%)	11 (50%)	24 (56%)
Current suicidal ideation [*N* (%)]	5 (24%)	10 (46%)	15 (35%)
Deliberate self‐harm (ever) [*N* (%)]	4 (19%)	8 (36%)	12 (28%)

ADIS CSR for SAD diagnosis, anxiety disorders interview schedule for children and adolescents clinical severity rating for social anxiety disorder diagnosis; *M*, mean; *SD*, standard deviation.

### Therapist activity

For OSCA completers (*n* = 20), therapists made 14.11 (*SD* = 2.93) telephone calls over the course of the 14‐week programme on average, lasting 24.02 min (*SD* = 4.23). They completed 1.11 webchats (*SD* = 0.33) lasting 62.44 min (*SD* = 11.40). Overall, the mean time spent in direct communication with participants was 398.67 min (6.65 h) (*SD* = 59.38).

### Patient activity on OSCA

In addition to the core modules, 7.20 (range: 5–11) optional modules were released. Patients logged onto OSCA for a total of 26.14 h (*SD* = 11.32) and logged 25.00 (*SD* = 10.75) completed behavioural experiments.

### Adverse events

No adverse events or serious adverse events were identified.

### Clinical outcomes

#### Primary outcomes

**Figure 2:**
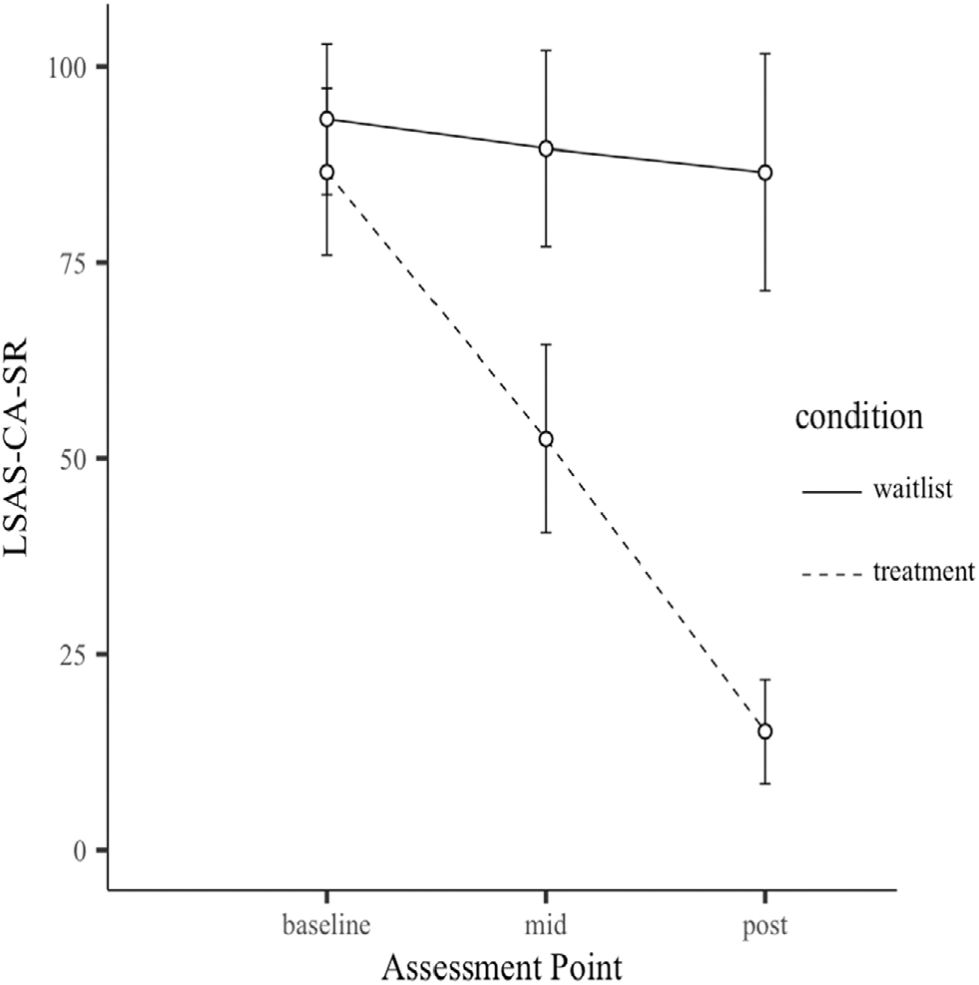
Mean LSAS‐CA‐SR scores for OSCA and waitlist at principal assessment points. Error bars represent 95% confidence intervals

Figure 2 shows LSAS‐CA‐SR by condition at key assessment points. Differences between arms was significant at mid and post, with OSCA showing superior effects. The between group effect size at post was large: 2.31 (Cohen's *d*; 95% CI: 1.85; 2.76). Table 2 reports unadjusted means and standard deviations, and Table 3 presents adjusted group differences and effect sizes.

**Table 2:** Unadjusted means and standard deviations for continuous outcome measures

Measure	Waitlist		OSCA	
	*M*	*SD*	*M*	*SD*
LSAS‐CA‐SR				
Baseline	93.29	22.43	86.59	25.43
Mid	89.55	28.54	52.48	28.08
Post	86.52	35.37	15.11	15.15
3 months fu	–	–	11.75	10.57
6 months fu	–	–	16.45	13.64
SPWSS				
Baseline	37.22	7.66	31.61	8.27
Mid	28.17	10.00	21.44	8.04
Post	32.50	9.23	14.12	5.43
3 months fu	–	–	10.75	6.96
6 months fu	–	–	12.60	7.36
SCQ – frequency				
Baseline	3.40	0.60	3.02	0.74
Mid	3.09	1.01	1.87	0.59
Post	3.62	0.88	1.25	0.31
3 months fu	–	–	1.22	0.27
6 months fu	–	–	1.28	0.41
SCQ – belief				
Baseline	58.18	19.82	53.96	19.12
Mid	51.28	20.98	23.02	14.95
Post	53.86	23.80	7.43	7.77
3 months fu	–	–	6.38	6.56
6 months fu	–	–	7.64	9.88
SBQ				
Baseline	1.48	0.18	1.45	0.13
Mid	1.77	0.18	1.02	0.47
Post	1.45	0.15	0.40	0.24
3 months fu	–	–	0.48	0.34
6 months fu	–	–	0.45	0.23
SAQ				
Baseline	3.26	0.92	3.39	0.71
Mid	3.45	1.12	4.08	0.89
Post	3.45	0.93	5.23	0.69
3 months fu	–	–	5.32	0.80
6 months fu	–	–	5.25	0.74
Concentration				
Baseline	3.67	2.01	4.50	1.71
Mid	4.00	1.81	5.19	1.94
Post	3.71	1.76	6.25	1.29
3 months fu	–	–	6.05	1.43
6 months fu	–	–	5.50	2.01
Participation				
Baseline	50.32	15.06	48.20	13.98
Mid	48.10	12.78	55.30	8.57
Post	48.73	16.07	69.26	8.22
3 months fu	–	–	68.85	9.29
6 months fu	–	–	66.70	11.73
Satisfaction				
Baseline	15.81	4.85	15.98	3.61
Mid	16.90	4.70	20.55	3.36
Post	18.41	5.05	22.63	3.50
3 months fu	–	–	22.45	3.46
6 months fu	–	–	21.80	3.43
SMFQ				
Baseline	13.81	7.22	13.59	7.01
Mid	13.86	8.29	7.05	4.97
Post	13.10	7.37	4.05	3.72
3 months fu	–	–	4.00	3.48
6 months fu	–	–	4.85	4.84
RCADS				
Baseline	70.86	23.02	66.09	20.15
Mid	–	–	–	–
Post	63.62	25.56	21.52	16.57
3 months fu	–	–	–	–
6 months fu	–	–	23.58	14.72
CALIS				
Baseline	19.57	6.61	16.59	8.18
Mid	–	–	–	–
Post	17.81	6.90	7.16	5.25
3 months fu	–	–	7.70	4.74
6 months fu			6.80	5.86
RCADS – parent				
Baseline	42.53	16.25	41.50	16.95
Mid	–	–	–	–
Post	52.99	17.13	29.09	14.86
6 months fu	–	–	–	–
CALIS – parent				
Baseline	22.00	10.79	20.11	11.50
Mid	–	–	–	–
Post	26.69	9.93	12.53	11.32
6 months fu	–	–	–	–

In the OSCA group, 22 participants provided complete data at baseline, 21 at mid‐treatment and 20 at post‐treatment. In the wait group, 21 participants provided data at baseline, 20 at mid‐treatment and 21 at post‐treatment. CALIS, Child Anxiety Life Interference Scale; LSAS‐CA‐SR, Liebowitz Social Anxiety Scale for children and adolescents – self‐report version; *M*, mean; RCADS, Revised Child Anxiety and Depression Scale (total scores); SAQ, Social Attitudes Questionnaire; SBQ, Social Behaviour Questionnaire; SCQ, Social Cognitions Questionnaire (mean scores); *SD*, standard deviation; SMFQ, Short Mood and Feelings Questionnaire; SPWSS, Social Phobia Weekly Summary Scale.

**Table 3:** Adjusted differences and effect sizes for continuous measures for intention to treat sample

Measure	Adjusted difference (*SE*) [95% CI], *p* value		Effect size Cohen's *d* [95% CI]			
	Mid	Post	Between group at mid	Between group at post	Within group pre‐post^a^	
					Treatment	Waitlist
LSAS‐CA‐SR	31.01 (6.29)	64.92 (6.29)	1.07	2.31	2.94	0.22
	[18.28, 43.74], *p* < .001	[52.19, 77.65], *p* < .001	[0.63, 1.51]	[1.86, 2.76]	[2.39, 3.49]	[−0.05, 0.50]
SPWSS	6.07 (2.63)	64.92 (6.29)	0.64	2.24	1.75	0.54
	[0.80, 11.34], *p* < .05	[52.19, 77.65], *p* < .001	[0.09, 1.22]	[1.57, 2.92]	[0.93, 2.57]	[−0.06, 1.03]
CASCQ – frequency	1.08 (0.21)	1.65 (0.22)	1.28	2.41	2.42	0.43
	[0.66, 1.53], *p* < .001	[1.21, 2.09], *p* < .001	[0.78, 1.78]	[1.77, 3.05]	[1.66, 3.16]	[−0.03, 0.83]
CASCQ‐ belief	25.29 (4.62)	42.98 (4.62)	1.36	2.34	2.75	0.19
	[16.04, 34.54], *p* < .001	[33.73, 52.23], *p* < .001	[0.86, 1.86]	[1.84, 2.85]	[2.09, 3.40]	[−0.12, 0.51]
CASAQ	−0.51 (0.21)	−1.52 (0.21)	0.53	1.99	2.29	0.20
	[−0.93, −0.09], *p* < .05	[−1.94, −1.10], *p* < .001	[0.09, 0.97]	[1.44, 2.55]	[1.70, 2.90]	[−0.11, 0.52]
CASBQ	0.71 (0.09)	1.01 (0.09)	1.93	4.95	5.22	0.18
	[0.53, 0.89], *p* < .001	[0.83, 1.19], *p* < .001	[1.44, 2.42]	[4.07, 5.83]	[4.25, 6.20]	[−0.94, 0.59]
Concentration	−0.89 (0.51)	−2.22 (0.51)	0.46	1.40	1.08	0.03
	[−1.91, 0.13], *p* = .09	[−3.24, −1.20], *p* < .001	[0.07, 0.99]	[0.76, 2.04]	[0.27, 1.89]	[−0.43, 0.49]
Participation	−6.65 (3.24)	−21.67 (3.20)	0.60	1.64	1.92	0.10
	[−0.16, −13.14], *p* < .05	[−15.26, −28.08], *p* < .001	[0.01, 1.18]	[1.16, 2.13]	[1.33, 2.51]	[−0.40, 0.21]
Satisfaction	−3.08 (1.14)	−3.91 (1.14)	0.74	0.87	1.09	0.51
	[−0.79, −5.37], *p* < .01	[−1.62, −6.20], *p* < .01	[0.19, 0.29]	[0.36, 1.39]	[0.42, 1.75]	[0.18, 0.85]
SMFQ	6.66 (1.29)	9.00 (1.29)	0.96	1.49	1.55	0.10
	[4.07, 9.25], *p* < .001	[6.41, 11.59], *p* < .001	[0.58, 1.33]	[1.06, 1.92]	[1.09, 2.01]	[−0.35, 0.16]
RCADS	–	35.44 (4.83)	–	1.60	1.87	0.29
		[25.66, 45.22], *p* < .001		[1.16, 2.04]	[1.35, 2.38]	[0.02, 0.56]
CALIS	–	7.41 (2.02)	–	1.18	1.04	0.25
		[3.32, 11.50], *p* < .001		[0.53, 1.82]	[0.45, 1.62]	[−0.16, 0.66]
Parent RCADS	–	22.56 (6.32)	–	1.37	1.32	0.39
		[9.87, 35.25, *p* < .001]		[0.60, 2.14]	[0.64, 2.00]	[−0.08, 0.87]
Parent CALIS	–	12.21 (4.12)	–	2.07	0.88	0.09
		[3.92, 20.50, *p* < .01]		[0.91, 3.23]	[0.37, 1.40]	[−0.33, 0.51]

In the OSCA group, 22 participants provided complete data at baseline, 21 at mid‐treatment and 20 at post‐treatment. In the wait group, 21 participants provided data at baseline, 20 at mid‐treatment and 21 at post‐treatment. All linear mixed effects models included baseline LSAS and gender as covariates, and a random effect of participant. The RCADS and CALIS were completed at baseline and post‐treatment/wait, not at mid‐treatment/wait. CALIS, Child Anxiety Life Interference Scale; CASAQ, Child & Adolescent Social Attitudes Questionnaire; CASBQ, Child & Adolescent Social Behaviour Questionnaire; CASCQ, Child & Adolescent Social Cognitions Questionnaire (mean scores); CI, confidence interval; LSAS‐CA‐SR, Liebowitz Social Anxiety Scale for Children and Adolescents – Self‐Report version; RCADS, Revised Child Anxiety and Depression Scale (total scores); *SE*, standard error; SMFQ, Short Mood and Feelings Questionnaire; SPWSS, Social Phobia Weekly Summary Scale.

^a^ Within‐group effect sizes obtained from separate linear mixed effects models including baseline score as a timepoint.

Two OSCA participants did not complete the diagnostic interview, so change in diagnostic status could not be demonstrated. Of the 22 OSCA participants, it could be demonstrated that 17 (77%) lost their SAD diagnosis post. Of the 21 waitlist participants, 3 (14%) lost their SAD diagnosis. Logistic regression indicated that condition was a significant predictor of diagnostic status, with the odds of losing SAD diagnosis over 20 times greater in the OSCA group (Table 4).

**Table 4:** Logistic regression to predict SAD diagnostic status at post

	Estimate (*SE*)	*Z*	*p*	Odds ratio [95%CI]
Intercept	−1.51 (1.36)	1.23	.267	0.22
Gender	0.31 (1.37)	0.23	.818	1.37 [0.09, 19.93]
Condition	3.02 (0.81)	3.75	<.001	20.54 [4.22, 99.88]

Overall model: χ^2^ (2) = 40.75, *p* < .001, *R* ^2^ = .35 (Cox & Snell), .47 (Nagelkerke). CI, confidence interval; *SE*, standard error.

#### Secondary outcomes

Significant group differences were found at post in favour of OSCA on all secondary self‐report measures (see Tables 2 and 3). Between group effect sizes ranged from 0.87 to 4.95. Significant group differences were found in favour of OSCA on parent‐reported internalising symptoms (RCADS) and impairment (CALIS), with between group effect sizes of 1.37 and 2.07, respectively (see Tables 2 and 3).

#### Follow‐up

**Figure 3:**
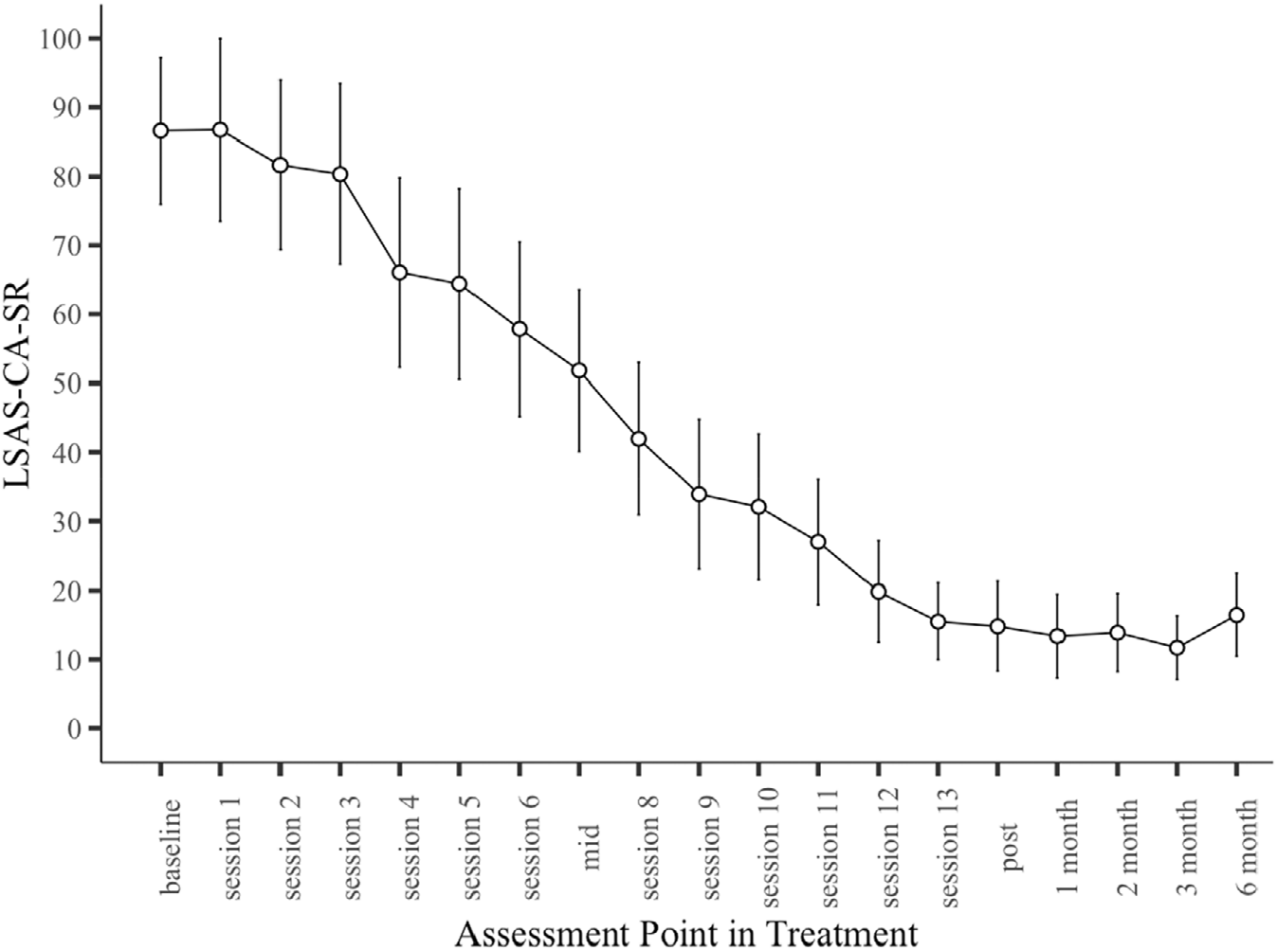
Mean LSAS‐CA‐SR scores weekly during treatment and at follow‐up assessments for the OSCA group. Error bars represent 95% confidence intervals

The OSCA group were followed up at 3‐ and 6‐months. Figure 3 shows LSAS‐CA‐SR scores through treatment and follow‐up. As can be seen in Table 2, gains were maintained, with linear mixed models showing a significant effect of time for all measures (*p* < .001). Pairwise comparisons revealed significant differences from baseline to post, 3‐month, and 6‐month (*p* < .001 for all measures) but not between post, 3‐month and 6‐month (*p* > .05 for all measures), suggesting that the substantial improvements were maintained at each follow‐up point. For RCADS, follow‐up data was only available at 6‐months. A significant effect of time was found, with pairwise comparisons indicating a significant difference between baseline and post and baseline and 6‐months (*p* < .001), but not between post and 6‐months (*p* > .05).

At post, it could be demonstrated that 77% of the OSCA group (17/22) no longer met criteria for DSM‐5 diagnosis of SAD. At the diagnostic assessment at 6‐months this had increased to 91%.

#### Therapeutic alliance and treatment credibility

Mean participant‐rated alliance was 75.00 (*SD* = 4.97) and mean therapist‐rated alliance was 69.25 (*SD* = 5.39). Credibility was also high, with a mean rating of 8.70 (*SD* = 0.91). In a linear regression, neither participant‐rated alliance (*b* = −0.126, *p* = .919), therapist‐rated alliance (*b* = 1.097, *p* = .205) nor credibility (*b* = −2.84, *p* = .662) predicted post‐treatment LSAS‐CA‐SR scores, controlling for baseline LSAS‐CA‐SR and gender.

### Exploratory mediation analysis

Results of exploratory analysis of candidate mediators of clinical improvement are presented in Table 5. The relationship between condition and LSAS‐CA‐SR post scores was mediated by mid‐point social anxiety‐related negative cognitions (frequency and belief ratings), attitudes and safety behaviours. Social anxiety‐related negative cognitions (belief) and safety behaviours showed the strongest mediation effect.

**Table 5:** Mediation of post scores

Mediator (at mid)	Total effect		direct effect		indirect effect		% Mediated
	Adjusted difference [95% CI]	*p*	Adjusted difference [95% CI]	*p*	Adjusted difference [95% CI]	*p*	
CASCQ‐f	−49.33	<.001	−36.02	<.001	−13.31	<.01	27
	[−60.14, −39.02]		[−49.58, −22.84]		[−22.99, −4.62]		
CASCQ‐b	−47.98	<.001	−28.46	<.01	−19.52	<.001	41
	[−58.38, −37.23]		[−42.26, −14.37]		[−31.43, −9.27]		
CASBQ	−47.77	<.001	−30.66	<.01	−17.11	<.01	36
	[−58.92, −35.32]		[−48.34, −12.49]		[−29.69, −3.78]		
CASAQ	−48.38	<.001	−40.54	<.001	−7.83	<.05	15
	[59.07, −37.19]		[−52.87, −28.33]		[−16.37, −0.40]		

CASAQ, Child & Adolescent Social Attitudes Questionnaire; CASBQ, Child & Adolescent Social Behaviour Questionnaire; CASCQ, Child & Adolescent Social Cognitions Questionnaire; CI, confidence interval.

## Discussion

This RCT examined the preliminary efficacy and mechanisms of action of Internet‐delivered therapist‐assisted Cognitive Therapy for SAD in adolescents (OSCA) in a sample of 43 young people recruited through schools. As we hypothesised, OSCA led to large improvements in social anxiety symptoms and a greater loss of SAD diagnosis post‐treatment compared to waitlist, as well as greater improvements in self‐and parent‐reported depression, anxiety and functioning. We were encouraged by the good preliminary support for the mediating role of cognitions and safety behaviours, consistent with the intended targets of CT‐SAD (Clark & Wells, 1995).

The between‐groups effect sizes for the trial were large and in line with earlier evaluations of face‐to‐face adolescent CT‐SAD. Ingul et al. (2014) reported a large effect [1.26 (95% CI: 0.55, 1.97)] of CT‐SAD compared to attention‐placebo. Likewise, two case series of face‐to‐face adolescent CT‐SAD (Leigh, Creswell, et al., 2021; Leigh & Clark, 2016) reported large pre‐ to post‐treatment improvements on the LSAS‐CA‐SR, with reductions of 82 and 56 points. In this study, OSCA was associated with a 72‐point drop. Whilst comparisons between studies with different samples and designs is difficult, these findings do suggest that outcomes from Internet‐delivered CT‐SAD may be comparable to those associated with face‐to‐face versions.

Encouragingly, findings from this study and those discussed above suggest that treatment approaches for adolescents based on Clark and Wells' model (Clark & Wells, 1995) are associated with clinically meaningful improvements. The processes specified in the model, cognitions, beliefs and safety behaviours, all showed improvements with OSCA compared to waitlist (*d*: 1.99–4.95) and all were found to mediate the observed effect of treatment on social anxiety.

As well as reductions in social anxiety, improvement was seen on all secondary outcome measures. The finding that an intervention that is fairly tightly focused on social anxiety can lead to more general improvements in mood and internalising symptoms is consistent with previous studies in adults (Cuijpers, Cristea, Weitz, Gentili, & Berking, 2016). Many adolescents presenting with SAD as a primary problem will have these associated difficulties (Garcia‐Lopez, Bonilla, & Muela‐Martinez, 2016), and so if replicated this finding has valuable clinical implications because it suggests that an additional separate depression intervention may not be needed for youth presenting with SAD and secondary depression. Improvements were also found on social participation, social satisfaction and anxiety‐related impairment indicating that OSCA is also associated with functional improvement. In addition, reported ability to concentrate in class showed improvements, which if replicated might suggest OSCA could have beneficial effects on academic as well as health‐related outcomes (Leigh, Chiu, & Clark, 2021).

With 20 of the 22 participants allocated to OSCA completing treatment, it appears that with fairly minimal adaptations, Internet‐delivered CT‐SAD can be delivered successfully to youth. Consistent with this, ratings of treatment credibility were high, as were therapeutic alliance ratings by both therapist and participant, despite the relatively brief remote therapist input. Alliance ratings were in line with those reported in other studies of Internet‐delivered psychological therapy for adolescent anxiety disorders (Anderson et al., 2012; Stjerneklar, Hougaard, & Thastum, 2019). However, neither credibility nor alliance ratings were predictive of outcome. This finding may be a result of the restricted range on the credibility and alliance scores, with all participants scoring high (Bland & Altman, 2011). An alternative explanation is that these aspects are not responsible for treatment gains. This suggestion is in keeping with other studies of Internet‐delivered CBT for anxiety (Stjerneklar et al., 2019) and findings from a meta‐analysis showing that alliance ratings collected early in the course of therapy are not associated with outcome (McLeod, 2011).

We recognise several limitations. Participants were actively recruited via schools rather than via routine clinic pathways. It is therefore possible that the sample does not represent adolescents seen in clinical settings. However, symptom severity at baseline [mean LSAS‐CA‐SR = 89.86 (*SD* = 23.97)] was similar to that reported in a study of adolescents with SAD recruited via CAMHS [mean LSAS‐CA‐SR = 97.41 (*SD* = 26.02)] (Leigh, Creswell, et al., 2021). Furthermore, baseline RCADS that were in line with those reported in an audit of UK CAMHS (Gibbons, Harrison, & Stallard, 2021). Comorbid diagnoses were observed in over half our sample, a third were experiencing suicidal ideation, and just under a third had engaged in deliberate self‐harm at some point in their lives. These characteristics suggest that our findings are relevant for youth referred to clinical services.

The sample was overwhelmingly female, despite recruiting from mixed schools. Although SAD is more prevalent in girls compared to boys (Asher, Asnaani, & Aderka, 2017), the discrepancy observed in the present sample is greater than one would expect. To examine the programme's efficacy with boys, future studies should seek a better gender balance. Future studies would benefit from examination of age as a possible moderator of treatment outcome. In addition, in future larger studies there will be value in understanding whether overall time spent on the OSCA programme, as well as use of particular features of the programme, predict clinical improvement. A further limitation is the use of a waitlist control condition, which does not permit examination of the relative contribution of specific and non‐specific treatment effects. The waitlist condition was deemed appropriate given this was the first trial of this intervention in adolescents, but an active comparison trial will be important. For example, it would be of particular use for clinical decision‐making to establish the comparative effects of OSCA and the current standard treatment for adolescent SAD (the generic form of CBT designed to treat all common anxiety presentations). The generalisability of the findings is also limited because the therapist assisting OSCA was a highly specialist clinical psychologist. The question of whether similar outcomes can be achieved by clinicians working in routine settings therefore needs to be addressed.

Findings from this preliminary study suggest that a therapist‐assisted Internet‐delivered version of CT‐SAD can be delivered to adolescents with high efficacy. If replicated, this could meaningfully improve outcomes for young people with SAD, for whom outcomes with the current standard treatment are disappointing. OSCA offers the potential of an accessible, effective intervention for this population and we hope our findings come at a good moment, with the global effort to increase provision of mental health interventions in schools (Patalay et al., 2016).

## Supporting information


**Table S1.** OSCA modules.
**Table S2.** Continuous outcome measures.
**Appendix S1.** COVID‐19 disruption.
