# Supplementary material for: Internet‐delivered therapist‐assisted cognitive therapy for adolescent social anxiety disorder (OSCA): a randomised controlled trial addressing preliminary efficacy and mechanisms of action
Source: J Child Psychol Psychiatry. 2022 Aug 9;64(1):145–55. doi: 10.1111/jcpp.13680 (PMC10087225; doi:10.1111/jcpp.13680)
Supplement: Supplementary file 1 — Table S1. OSCA modules. Table S2. Continuous outcome measures. Appendix S1. COVID‐19 disruption. [file JCPP-64-145-s001.docx]

**Supporting Information**

Table S1. OSCA Modules

| Core Modules (allocated to all users) |
| --- |
| Introducing the treatment |
| Getting started |
| Feeling self-conscious |
| Safety behaviours |
| Attention & safety behaviours experiment |
| Watching your conversation videos |
| Getting out of your head and into the world |
| Behavioural experiments |
| Additional Modules (for particular fearful concerns and problems) |
| Blushing |
| Shaking |
| Sweating |
| Having conversations |
| Feeling boring |
| Feeling stupid |
| Feeling responsible for others enjoyment |
| Decatastrophizing |
| Worrying in advance |
| Going over social situations after they’ve happened |
| Leaving the past behind |
| Managing my inner critic |
| Self-esteem |
| Managing my mood |
| Giving myself credit |
| My therapy blueprint |
| \| Modules for to help patients prepare for follow-up sessions \| \| --- \| |
| Preparing for first follow-up |
| Preparing for second follow-up |
| Preparing for third follow-up |

Table S2 Continuous Outcome Measures

| **Scale** | **Rater** | **Measurement points** | **Reliability in study sample (Cronbach’s α)** |
| --- | --- | --- | --- |
| Liebowitz Social Anxiety Scale – Children & Adolescents – Self-report (LSAS-CA-SR) | self | Pre, mid, post  (OSCA arm: weekly through treatment and at 1-, 2-, 3-, and 6-months post) | α = .94 (baseline)  α = .99 (post) |
| Social Phobia Weekly Summary Scale | self | Pre, mid, post  (OSCA arm: weekly through treatment and at 1-, 2-, 3-, and 6-months post) | α = .78 (baseline)  α = .89 (post). |
| Revised Child Anxiety & Depression Scale (RCADS) | self  parent | Pre, post | Self-report:  α = .94 (baseline)  α = .98 (post)  Parent-report:  α = .92 (baseline)  α =.96 (post) |
| Short Mood and Feelings Questionnaire (SMFQ) | self | Pre, mid, post  (OSCA arm: weekly through treatment and at 1-, 2-, 3-, and 6-months post) | α = .91 (baseline)  α = .94 (post) |
| Peer Victimisation Scale | self | Pre, mid, post | α = .71 (baseline)  α = .48 (post) |
| Social Satisfaction Scale | self | Pre, mid, post | α = .62 (baseline)  α = .81 (post) |
| Social Participation Questionnaire | self | Pre, mid, post | α = .87 (baseline)  α = .94 (post) |
| Child Anxiety Life Interference Scale (CALIS) | self  parent | Pre, post | Self-report:  α = .85 (baseline)  α = .92 (post)  Parent-report:  α = .88 (baseline)  α = .85 (post) |
| Child & Adolescent Social Cognitions Questionnaire (CASCQ) | self | Pre, mid, post  (OSCA arm: weekly through treatment and at 1-, 2-, 3-, and 6-months post) | Frequency ratings:  α = .91 (baseline)  α = .98 (post)  Belief ratings:  α = .94 (baseline)  α = .98 (post) |
| Child & Adolescent Safety Behaviours Questionnaire (CASBQ) | self | Pre, mid, post  (OSCA arm: weekly through treatment and at 1-, 2-, 3-, and 6-months post) |  |
| Child and Adolescent Social Attitudes Questionnaire (CASAQ) | self | Pre, mid, post | α = .93 (baseline)  α = .97 (post) |
| Working Alliance Inventory (WAI) | self  therapist | Week 2 of OSCA | Self-report:  α = .82  Therapist-report:  α = .85 |
| Credibility of Therapy Scale | self | Week 2 of OSCA | α = .83 |

### Appendix S1. COVID-19 Disruption

In response to the COVID-19 pandemic, the Government of England took the decision to close all secondary schools from 20^th^ March 2020 except to an exceptional minority of children. Schools reopened in a limited fashion in June 2020 and then fully in September 2020, with additional measures such as mask-wearing and year group ‘bubbles’ (in which social contact was limited to within year groups). Schools closed again in January 2021 and reopened in March 2021. As well as the restrictions placed on schools, the Government of England also imposed restrictions on travel, socialising, and attending hospitality, entertainment, and retail venues. Requirements to wear a face mask and maintain a social distance were also introduced. The pandemic-related restrictions and closures affected the trial in several ways. The trial was suspended from March 2020 – September 2020 during the first school closure. This meant that the wave of school screening that was due to commence in late March 2020 to recruit participants was cancelled. Despite the lockdowns, all trial participants received a full course of treatment. The two OSCA patients in active treatment when the first lockdown was imposed were at session 13 and session 14 of the programme and so they had completed all or almost all of therapy. For those on the waitlist (n=5) at that point, post-wait treatment was delayed until the trial recommenced. Pandemic related restrictions also led to all assessments moving online to video calls. For patients receiving treatment whilst COVID restrictions were in place, the therapist ensured that suggestions (e.g., for behavioural experiments) were always in line with current Government guidelines.
